# Longitudinal Change in Gait and Motor Function in Pre-manifest Huntington’s Disease

**DOI:** 10.1371/currents.RRN1268

**Published:** 2011-10-04

**Authors:** Ashwini K. Rao, Pietro Mazzoni, Paula Wasserman, Karen Marder

**Affiliations:** ^*^Program in Physical Therapy, Department of Rehabilitation and Regenerative Medicine and Sergievsky Center, Columbia University College of Physicians and Surgeons. New York, NY. USA; ^†^Motor Performance Lab, Dept. of Neurology, Columbia University, New York; ^‡^Department of Neurology, G.H. Sergievsky Center at the Columbia University College of Physicians and Surgeons, New York, NY and ^§^Department of Neurology, Psychiatry, Sergievsky Center, and the Taub Institute for the Aging Brain, Columbia University College of Physicians and Surgeons

## Abstract

The purpose of this study was to examine longitudinal change in gait and motor function in pre-manifest Huntington’s disease (HD).

We examined ten pre-manifest subjects at baseline, one and five years. Quantitative gait data were collected with an electronic mat (GAITRite®). We analyzed measures related to speed (velocity, step length, cadence), asymmetry (step length difference), dynamic balance (percent time in double support, support base) and variability in stride length and swing time. Motor function was assessed with the motor component of the Unified Huntington’s Disease Rating Scale.

Gait velocity decreased (p=0.001), whereas step length difference (p=0.006), stride length variability (p=0.0001) and swing time variability increased (p=0.0001) from baseline to year five. Step length difference (p<0.05) and swing time variability (p<0.05) increased marginally in one year from baseline. UHDRS Total motor score increased over five years (p=0.003), though the increase in one year was not significant (p=0.053). Of the individual motor domain scores (eye, hand movements, gait and balance, chorea) only dystonia worsened over five years (p=0.02). Total motor score (r2= 0.49, p<0.001) and swing time variability (r2= 0.22, p<0.009) were correlated with estimated years to diagnosis.

Our results present the longest longitudinal follow up of gait in pre-manifest HD thus far. Despite the small sample size, quantitative gait analysis was able to detect changes in gait speed, symmetry and variability. Swing time variability was particularly important because it increased in one year from baseline and was correlated with estimated time to diagnosis. Our results highlight the importance of predictive outcomes such as gait variability using quantitative analysis.

## **Introduction**

      Huntington’s disease (HD) is an autosomal dominant disorder characterized by motor, cognitive and behavioral impairments that worsen over time.^[1][2][3]^ The diagnosis of HD is based on the presence of clinically observable motor impairments.^[4]^ However, recent work has demonstrated that motor, cognitive and behavioral impairments can be detected in pre-manifest individuals well before diagnosis^[1][3][5][6][7][8][9][10]^. 

      Cross-sectional gait analysis has shown that bradykinesia (decreased velocity and stride length), impaired dynamic balance (increased time in double support) and high gait variability (stride length, step time and swing time) are seen in pre-manifest HD.^[9]^ In symptomatic HD, the aforementioned impairments worsen and are accompanied by additional impairments, such as decreased cadence and stride length, and increased support base.^[11][12]^ Worsening of gait and balance impairments over the course of the disease eventually contribute to falls and to the need for institutional care.^[13], [14]^

      While cross-sectional analysis highlighted the sensitivity of quantitative gait measures across the spectrum of disease, longitudinal analysis is necessary to determine which aspects of gait change with time. Knowledge of gait parameters sensitive to change over time will be important for future clinical trials aimed at delaying disease onset and for identifying targets for therapeutic intervention.

      The purpose of this study was to examine which aspects of motor function (gait and UHDRS motor scores) change over time and examine if changes in motor function are correlated with estimated time of diagnosis. We report longitudinal analysis for a subset of subjects from the original cross-sectional study^[9]^ tested on three occasions over a five-year period.  

## **Methods**

### Subjects 

      Ten pre-manifest HD subjects (5 females) were recruited from subjects enrolled in PREDICT-HD, a multi-center collaborative study that examines neurobiological predictors of HD^[1]^. Subjects were tested on the same day as their PREDICT-HD testing. The local Institutional Review Board approved study procedures and all subjects provided written informed consent before participation in accordance with the Declaration of Helsinski. Subjects were tested by a movement disorder specialist and were recruited in the study if they had genetic confirmation and did not meet the criteria for clinical diagnosis of HD.^[1][15] ^ At study entry mean age of subjects was 38.6 years (SD, 7.94, Range= 27-54), mean height was 1.71 m (SD= 0.11) and weight was 73.85 kg (SD= 14.62).

      From fifteen subjects tested in our cross-sectional study, we followed ten subjects longitudinally.^[9]^ Two of the fifteen subjects dropped out and three others had missing gait data and thus were not included in the analysis. The remaining ten subjects were tested three times within a five-year period: at baseline, one year and five years. None of the subjects were taking neuroleptic medications and none reported any orthopedic or neurological illness that would impair walking.

### Quantitative Gait Assessment

      Details of the quantitative gait assessment have been published previously.^[9]^ Briefly, spatio-temporal gait data were recorded with the GAITRite^®^ (4.6 m long mat with pressure sensors embedded in its surface). At each visit subjects were asked to walk at a preferred speed along a 10-meter long hallway free of distractions. The starting position for each trial was 3 m from the beginning of the mat and termination was 3 m from the end of the mat in order to remove transient effects of gait initiation and termination. Subjects were given a practice trial before data collection.

      We analyzed measures related to gait speed (velocity, step length and cadence), gait asymmetry (step length difference), dynamic balance (double support time and support base) and gait variability (stride length and swing time coefficient of variation). The dependent variables were chosen based on prior cross-sectional analysis in subjects with pre-manifest and manifest HD.^[9][16]^

### Clinical Assessment

      A movement disorder specialist rated the motor items of the UHDRS (ranging from 0-124, higher numbers indicating greater impairments).^[17]^ A diagnostic confidence rating was assigned on the basis of the total motor score, as follows: 0, no abnormalities; 1, non-specific motor abnormalities (<50% confidence); 2, motor abnormalities that may be signs of HD (50-89% confidence); 3, motor abnormalities that are likely signs of HD (90-98% confidence); 4, motor abnormalities that are unequivocal signs of HD (>99% confidence).^[15]^

      From the UHDRS items we analyzed the total motor score and individual domain scores (chosen as in prior work)^[4], [18]^ as follows: UHDRS eye movement score which was computed as the sum of items 1-3; UHDRS hand movement score, which was computed as the sum of items 6-8; UHDRS dystonia score, which was the sum of items 11 A-E; UHDRS Chorea score, which was the sum of items 12 A-G; and UHDRS gait and balance score, which was the sum of items 13-15. For all individual UHDRS domain scores higher scores indicate greater impairments. We analyzed the motor items of the UHDRS to compare their sensitivity to change over time with that of the gait outcome measures.

### Data Analysis

We conducted two analyses:  

1) Longitudinal change over three visits was evaluated by conducting repeated measures analysis of variance for quantitative gait measures and UHDRS (total motor score and individual domains) with visit as the independent variable. We analyzed individual domains of eye movements (sum of items 1-3), hand movements (sum of items 6-8), dystonia (sum of items 11 a-e), chorea (sum of items 12 a-g), gait and balance (sum of items 13-15).^[4][18]^ Level of significance was adjusted to 0.01 to account for multiple comparisons. Any significance values between 0.05 and 0.01 were considered to be of marginal interest. Post-hoc comparison (Tukey’s Honestly Significant Difference) was used to evaluate change from baseline to year 1 for gait and UHDRS motor variables that demonstrated significant main effects.

2) Predictive validity: Variables that significantly changed over time in repeated measures analysis were then entered into a linear regression analysis with estimated years to diagnosis as the dependent variable. Estimated years to diagnosis was computed from a parametric survival model that used CAG repeat length and the person’s age at the time of motor testing.^[19]^ A blinded statistician from the PREDICT-HD study^[1]^ performed the computations for predicted years to diagnosis. Statistical analyses were conducted in SPSS (version 16.0) and results are presented as mean + standard deviation.  

## **Results**

### Longitudinal Change over time

      We first conducted repeated measures analysis to evaluate whether quantitative gait measures and Unified Huntington’s disease Rating Scale (UHDRS) items changed over time. Results of the repeated measures analysis for quantitative gait measures are shown in Table 1. Among speed related variables gait velocity decreased in five years (F=10.15, p=0.001; significant linear trend = 0.007). Post-hoc analysis demonstrated that the change from baseline to year 1 was not significant. Gait asymmetry, measured by step length difference, increased (F=6.83, p=0.006, significant linear trend = 0.016). The change from baseline to year 1 was marginally significant (0.01< p <0.05). Gait variability demonstrated the largest change; stride length coefficient of variation increased in 5 years (F=15.85, p<0.0001, significant linear trend=0.001). However, the change from baseline to year 1 was not significant (p=0.28). Swing time coefficient of variation significantly increased in 5 years (F= 13.31, p<0.0001, significant linear trend= 0.002). Post-hoc analysis demonstrated that the change from baseline to year 1 was of marginal significance (0.01< p <0.05).  None of the other gait measures changed significantly in 5 years. To summarize, step length difference (0.01< p <0.05) and swing time coefficient of variation (0.01< p <0.05) changed marginally in one year from baseline.

Table 1: Analysis of Variance with score at baseline, year 5, and significance value for quantitative gait measures   

**Table:** 

Outcome	Score at Baseline	Score at Year 5	F value	p-value
Velocity (m/sec) + SD	1.39 + 0.06	1.18 + 0.1	**10.15**	**0.001**
Cadence (steps/min) + SD	120.95 + 4.84	114.97 + 15.97	2.35	0.124
Step length (m) + SD	0.69 + 0.016	0.61 + 0.09	3.13	.068
Step length difference (m) + SD	.024 + 0.597	0.18 + 0.17	**6.83**	**0.006**
Double support time (sec) + SD	0.27 + 0.008	0.29 + 0.06	1.15	0.339
Support base + SD	0.06 + 0.98	0.08 + 0.03	1.11	0.352
Stride length Cov + SD	2.41 + 0.514	10.49 + 4.8	**15.85**	**0.0001**
Swing time Cov + SD	3.32 + 0.21	14.06 + 8.06	**13.31**	**0.0001**

   SD, standard deviation cov, coefficient of variation;

      Table 2 shows the results of the repeated measures analysis for UHDRS motor items, including total motor score, diagnostic confidence and five motor domain scores. At baseline pre-manifest (pHD) subjects, on average, demonstrated minor non-specific motor abnormalities based on their total motor score of 3.5 + 1.78 and diagnostic confidence rating of 1.0 + 0.47. Five years from baseline, total motor score increased to 10.1 + 2.1 (range 4-19): while there were four subjects with a total motor score above 10, none of them was given a diagnosis confidence rating of 4 (indicating unequivocal signs of HD) because none of the individual items scored more than 2 out of a total of 4. While total motor score changed across five years (p= 0.003), there was no significant difference between baseline and one year (p = 0.06). Dystonia and chorea scores increased across visits, though only dystonia was marginally significant (p=0.028). None of the individual UHDRS motor domain scores changed significantly over five years (Table 2).

- Table 2: Analysis of Variance with score at baseline, year 5, significance value for items from UHDRS scale 

**Table:** 

Outcome	Baseline Score	Score at Year 5	F	p-value
Total Motor Score + SD	3.5 + 1.78	10.1 + 2.1	**9.36**	**0.003**
Diagnostic Confidence + SD	1.0 + 0.47	1.4 + 0.84	1.34	0.265
UHDRS eye movements + SD	0.6 + 0.69	1.5 + 1.5	2.39	0.12
UHDRS hand movements + SD	1.7 + 1.25	3.5 + 2.63	2.83	0.08
UHDRS chorea + SD	0.2 + 0.63	1.9 + 2.37	3.48	0.052
UHDRS dystonia + SD	0.2 + 0.42	1.0 + 1.05	4.36	0.028
UHDRS gait and balance + SD	0.1 + 0.32	0.3 + 0.48	0.78	0.47

UHDRS, Unified huntington’s disease rating scale; SD, standard deviation

### Predictive validity

      Variables that demonstrated significant change over five years (gait velocity, step length difference, stride length and swing time variability and UHDRS total motor score) were entered into linear regression analysis with estimated years to diagnosis as the independent variable. We chose linear regression because initial repeated measures analysis demonstrated a significant linear trend for all variables. Only total motor score (r^2^=0.489, p<0.0001), and swing time coefficient of variation (r^2^=0.22, p<0.009) were significant predictors of estimated years to diagnosis, as shown in Figure 1. 

**Figure:**
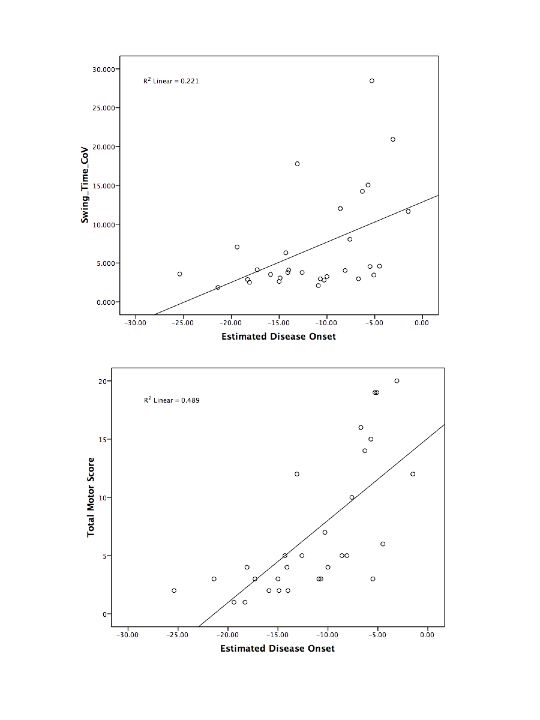
Swing time variability (top) and total motor score (bottom) plotted as a function of estimated disease onset. Each subject has three data points corresponding to baseline, year 1 and year 5.  Each graph includes the regression line and r^2^ value.

## **Discussion**

      Quantitative longitudinal analysis demonstrates that gait impairments worsen over a period of five years in pre-manifest Huntington’s disease (HD). Gait velocity decreased and gait asymmetry increased over a five-year period. Gait variability (stride length and swing time coefficient of variation) demonstrated the greatest changes, (Table 2). Gait asymmetry and swing time coefficient of variation were marginally higher one year from baseline indicating that changes in these measures can be detected in one year. 

      In contrast, measures related to dynamic balance (such as double support time) did not change despite the fact that cross-sectional analysis previously demonstrated impairments in pre-manifest HD, which worsened in manifest HD.^[9]^ Increase in double support time is interpreted as a compensatory response to impaired balance.^[20]^ It is possible that double support time did not change because balance impairments may remain subtle in the pre-manifest stage, and increase in a step-like manner in manifest HD. This is borne out by the fact that double support time for pre-manifest HD subjects was not different from stage I manifest HD subjects in cross-sectional analysis.^[9]^

      Prior cross-sectional analysis also demonstrated that gait measures have good sensitivity and specificity, highlighting their importance as good markers of disease onset.^[9]^ Here we extend those results to demonstrate the importance of quantitative gait measures, particularly gait variability, as good markers of disease progression. Since motor variability is higher in pre-manifest HD across modalities (eye movements,^[21]^ arm movements,^[22]^ finger tapping^[23][24]^ and precision grip^[6][10]^) and is predictive of estimated diagnosis, this strongly suggests that motor variability is an early putative deficit in HD rather than simply a generalized feature of impaired motor control.

      Clinical motor assessment revealed that UHDRS total motor score increased over the five-year period, indicating worsening of motor impairments. The change in total motor score was not due to worsening impairments in any specific domain (such as eye movement, hand movement, gait and balance) but was a reflection of generalized worsening of motor function. It was interesting to note that the gait and balance section of the UHDRS was unable to detect impairments detected by quantitative analysis, presumably because of the lack of sensitivity of clinical assessment. 

While four of ten subjects had a total motor score greater than 10 five years from baseline, none of them received a score of 4 on diagnostic confidence item of the UHDRS. These subjects demonstrated very subtle impairments across motor domains rather than significant impairments in any domain that may have constituted grounds for diagnosis. Change in total motor score was not significant in one year from baseline. Results of the clinical assessment are in contrast with quantitative gait assessment, which allowed for the detection of specific changes in gait velocity, symmetry and gait variability over time. The increased sensitivity of quantitative gait assessment compared with clinical assessment in detecting specific impairments will be important in designing targeted therapeutic intervention. While total motor score is very useful in clinical assessment of motor function for the purpose of diagnosis, quantitative gait measures will be important as outcomes in clinical trials in pre-manifest and very early manifest HD.

      Despite the small sample size, our study has the longest longitudinal follow up thus far for pre-manifest HD. The one-year follow up results from the Track-HD study are the only other available longitudinal data.^[25]^ In the Track-HD study, total motor score changed significantly in pre-manifest and manifest HD in one year compared with controls. The small sample size in our study may explain why we did not find significant change in total motor score in one year.  Interestingly, the Track-HD study did not find any changes in gait variability (or grip and tongue force variability) over a one year period despite the fact that these measures were impaired in pre-manifest HD in cross sectional analysis.^[3]^

      In conclusion, the findings that within-subject gait variability increased, and that this increase was correlated with estimated diagnosis, underscore the utility of quantitative gait measures in pre-manifest HD. In the future, it will be important to replicate these results in a larger sample. Future therapeutic trials will benefit from sensitive measures of disease progression, and gait analysis may provide such a measure. Design of future trials may also necessitate clarification of pathological mechanisms underlying gait impairments in order to identify modifiable targets for intervention.  

**Author Contributions**

*Ashwini K. Rao*: Research project: conception, organization and execution; statistical analyses: design and execution; manuscript writing: writing the first draft and making subsequent revisions.

*Pietro Mazzoni*: Research project: organization and execution; statistical analyses: review; manuscript writing: making subsequent revisions.

*Paula Wasserman*: Research project: organization and execution; statistical analyses: review; manuscript writing: making subsequent revisions.

*Karen S. Marder*: Research project: conception and organization; statistical analyses: review; manuscript writing: making subsequent revisions. 

**Statistical Analyses:** Dr. Rao conducted the statistical analyses.

**Acknowledgements: ** We acknowledge all pre-manifest subjects for participating in the study, Jane Paulsen, PhD and James Mills, PhD for providing access to PREDICT-HD data. 

**Funding:** This work was partially funded by Huntington’s Disease Society of America Investigator grant (AR), National Institute of Health grant (Paulsen 5R01NS040068-10) and Eunice Kennedy Shriver National Institute of Child Health and Human Development grant (AR- K01-HD060912).

**Competing Interests:** The authors report no competing interests. 

**Correspondence: **Dr. Ashwini K. Rao, Neurological Institute, 8^th^ Floor, Columbia University, 710 West 168th Street, New York, NY, 10032, USA. 

Tel: (212) 305 - 1647, FAX: (212) 305 -4569, Email: akr7@columbia.edu
